# CAR-T in relapsed refractory high-grade glioma and glioblastoma – who, what, when and how?

**DOI:** 10.3332/ecancer.2025.2001

**Published:** 2025-09-30

**Authors:** Deevyashali Parekh, Ansy H Patel, Areeb Khan, Eloho Olojakpoke, Ashay Karpe, Zoya Peelay, Vijay Patil

**Affiliations:** 1 Department of Medicine, SUNY Upstate Medical University, Syracuse, NY 13210, USA; 2 Department of Medical Oncology, Sunrise Oncology Center, Mumbai 400092, India; 3 Department of Medical Oncology, Sunact Cancer Institute, Thane 400615, India

**Keywords:** receptors, chimeric antigen, glioblastoma, epidermal growth factor receptor VIII, antigens, neoplasm, CD28 antigens, single-chain antibodies, granzymes, T-lymphocytes, cytokines, matrix metalloproteinase 2, tumour microenvironment, glioma, treatment outcome, central nervous system, cell- and tissue-based therapy

## Abstract

Recurrent high-grade gliomas have a dismal prognosis. This review article aimed to explore and help answer the questions about which group of patients would benefit from chimeric antigen receptor therapy (CAR-T) cell therapy in this setting, the timing of intervention and the therapeutic efficacy. CAR-T cell therapy involves the extraction of T-cells from patients, genetic modification of these cells to express chimeric antigen receptors on their cell surface, which are selectively targeted towards tumour-expressed antigens and a procedure of immune-depletion followed by re-introducing these engineered CAR-T cells into the host via infusion. Gliomas, particularly glioblastoma, present unique challenges due to their immune-evasive nature, location within the central nervous system and antigenic heterogeneity. Thus, several potential antigenic targets are being explored for CAR-T cell therapy, including B7 homolog 3, Disiloganglioside, Eph-A2, Eph-A3, IL-13Ra2, HER2, EGFRvIII and Matrix metalloproteinase-2.

## Introduction

Recurrent high-grade glioma and glioblastoma have a dismal prognosis. No standard of care systemic therapy has been established for these tumours. The National Comprehensive Cancer Network guidelines would suggest the use of Lomustine (CCNU), temozolomide or Procarbazine, CCNU and Vincristine in this situation. The use of Bevacizumab, an antiangiogenic agent, in this situation is described in the literature, with promising results being reported in a phase 2 study [1] (BELOB). However, the agent failed to make its mark in the phase 3 randomised study [2] (EORTC 26101). In this study, the addition of Bevacizumab to CCNU marginally improved the median progression-free survival (PFS) by 2.7 months, but there was no improvement in quality of life or overall survival (OS). Thus, at present, there is a large scope for improvement in the prognosis of these recurrent-refractory high-grade glioma and glioblastoma.

Cellular therapies like Chimeric antigen receptor therapy (CAR-T) have established themselves as the standard of care therapies in CD-19 positive B cell lymphomas and leukaemias and B-cell maturation antigen CAR-T has established itself in Multiple Myeloma. Solid tumour CAR-T has been showing promising results in phase 1 and phase 2 studies. CAR-T innovation enables us to, theoretically, do systemic targeted elimination of cancer cells in a major histocompatibility complex independent manner. However, the brain is considered an immune-privileged organ, and whether CAR-T would be able to have similar efficacy as haematological tumours in this tumour is an open question. Hence, this review article was planned by the authors with the aim of answering the questions of who, when, what and how CAR-T can benefit high-grade glioma or glioblastoma patients from a clinician's perspective.

## Methods

The authors performed an advanced PubMed search on 1st March 2025 with the following keywords

1. ‘CAR T’[All Fields] AND ‘Glioma’[All Fields]
2. ‘CAR T’[All Fields] AND ‘Glioblastoma’[All Fields]

The filter applied in the process was those articles published in the last 5 years. We came across 195 and 233 articles, respectively, with the two keyword searches. Then, manually among these articles, unique articles were selected, subjected to below-mentioned criteria

1. Human research
2. Case report or phase 1 or phase 2 or phase 3 articles.

**Figure 1.**
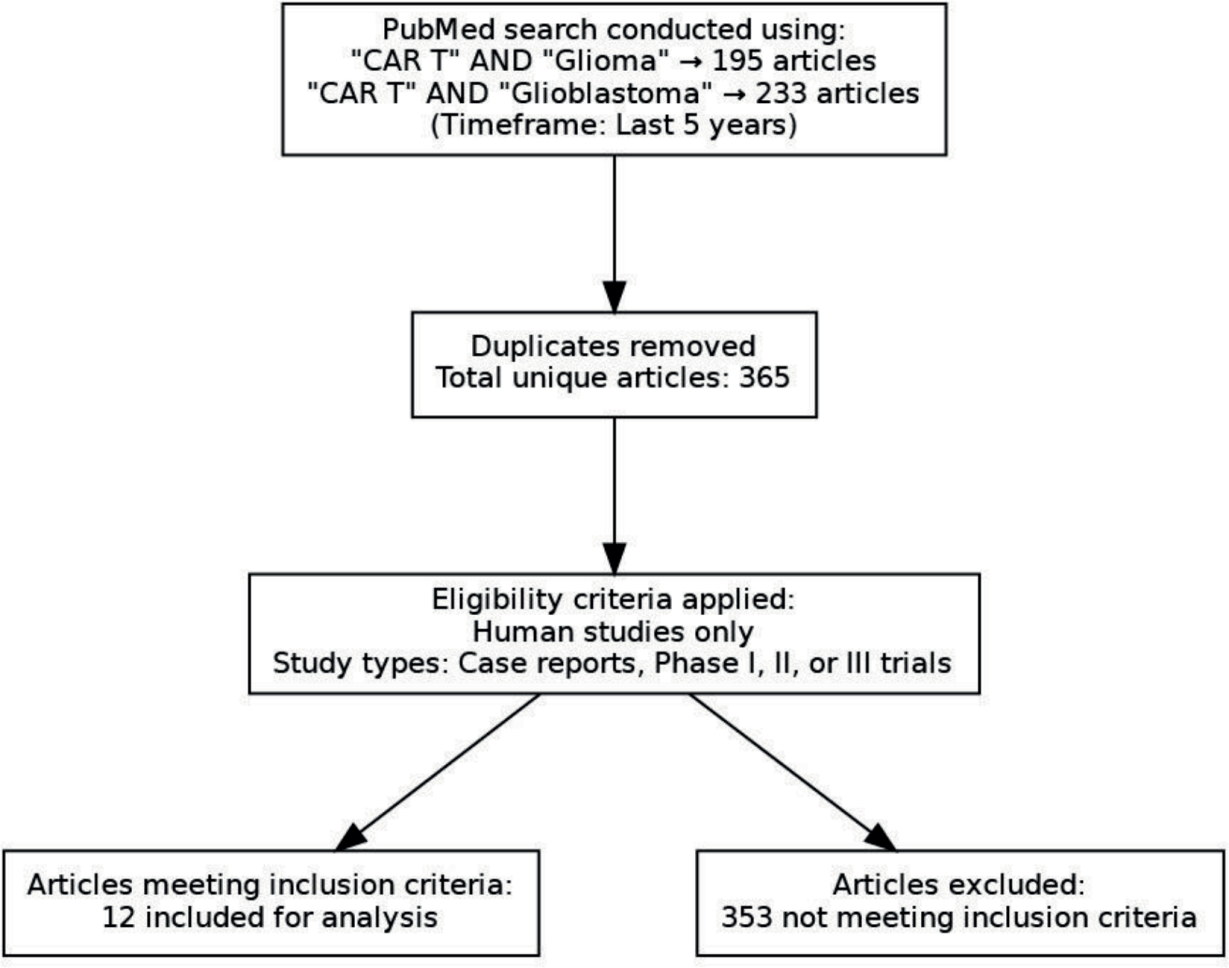
Flowchart of search strategy and article selection.

**Figure 2.**
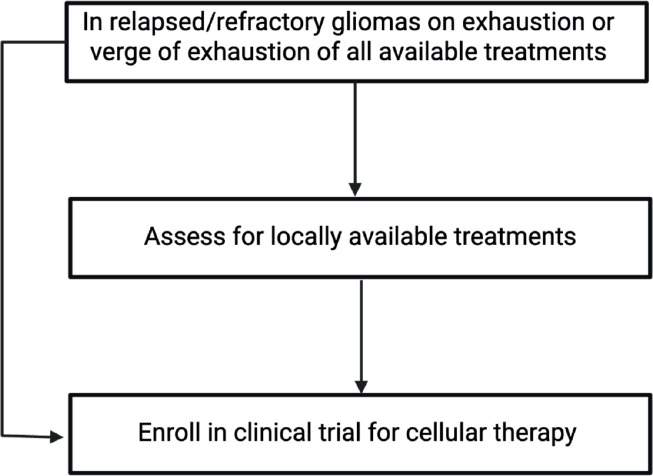
Algorithm for treatment assessment of r/r glioma patients.

We identified 365 unique articles, and these articles were used to answer the questions about CAR-T in whom, when, what and how from the clinician's perspective. The question of who and when was answered by looking at the inclusion and exclusion criteria in the studies. The question of what and how took into account the CAR-T details, its antigen and method of delivery. This is depicted in Figure 1. In addition, the efficacy of these therapies and adverse events were collated. In addition, an algorithm for how to use this knowledge in clinics has also been provided in Figure 2.

## Discussion

While our study aims to answer the clinical questions about the patient population, CAR-T selection and timing for CAR-T use in gliomas, it is important to understand the underlying mechanism of how CAR-T therapy works and the specific targets being developed and used in clinical studies.

CAR-T cells are a relatively novel immune-mediated therapy developed in an attempt to overcome factors like dependence on host immunogenicity, immune-escape by tumours, heterogeneity in the tumour microenvironment (TME), leading to reduced efficacy and factors leading to resistance against conventional chemotherapeutic strategies. CAR-T cell therapy involves the extraction of T-cells from patients, genetic modification of these cells to express chimeric antigen receptors (CARs) on their cell surface, which are selectively targeted towards tumour-expressed antigens and a procedure of immune-depletion followed by re-introducing these engineered CAR-T cells into the host via infusion.

CAR-T cells are broadly engineered with three key domains. An extracellular antigen recognition domain, a transmembrane domain responsible for downstream activation and intracellular domains responsible for the induction of the anti-tumour response. The extracellular domain typically consists of a single-chain variable fragment (scFv) that is derived from an antibody against the targeted tumour-specific antigen with the function of selective antigen-recognition. The transmembrane and intracellular domains are responsible for downstream signalling and activation of T-cells, release of cytotoxic granzymes and inflammatory cytokines for the anti-tumour activity. Modifications in subsequent generations of CAR-T cell therapy is generally within these two domains and aim to produce greater longevity of action, amplification of immune effector cells and/or improved therapeutic efficacy.

**Figure 3.**
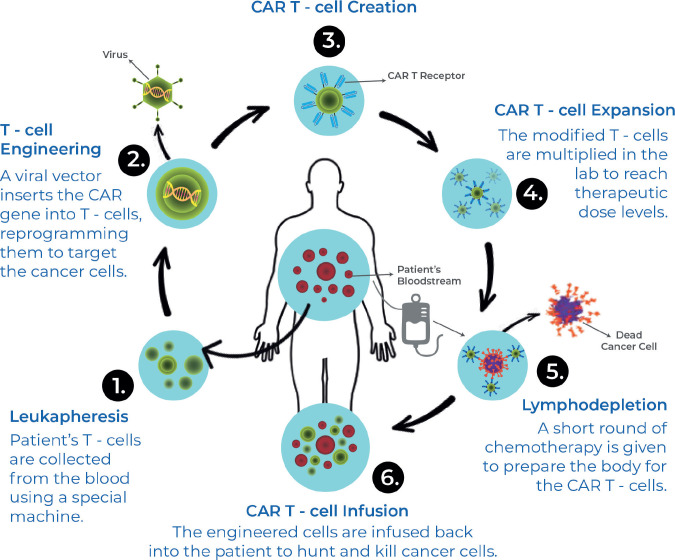
CAR-T therapy production process.

**Figure 4:**
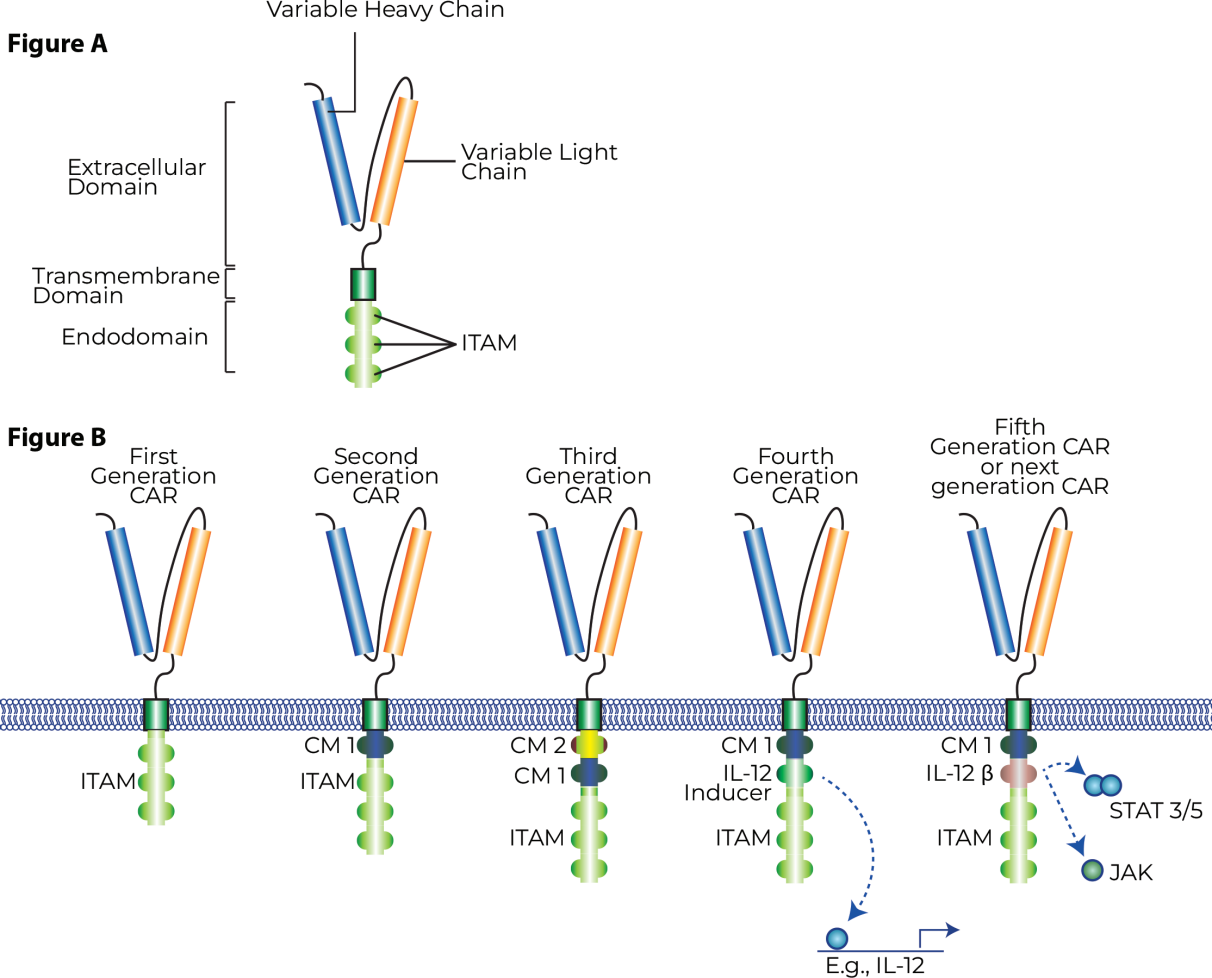
(A): General structure of a chimeric antigen receptor T-cell. (B): Generations of CAR-T cells.

First-generation CAR-T cells consist of a scFv domain, a transmembrane domain and an intracellular CD3-signaling domain. The first-generation CAR-Ts had a relatively lower duration of action and clinical efficacy. Second-generation CAR-T cells incorporated a costimulatory domain like CD28 OR 4-1BB in an effort to increase the duration of effect. Third-generation CAR-T cells were able to simultaneously incorporate these costimulatory domains, like CD28 and 4-1BB, sometimes OX-40 to further increase the duration of effect. Recently, a fourth generation of CAR-T cells is being worked on, which is being termed an ‘armoured CAR-T cell’ or ‘TRUCK-T’, which supplements a third-generation CAR-T cell with surface-bound cytokines acting as a third signal and serving to modulate the TME or enhance duration of action and efficacy [3]. Recent data in animal models has shown how the addition of these pro-inflammatory cytokines significantly improves the efficacy of CAR-T cells even in high-grade gliomas [4]. Figure 3 depicts CAR-T structure and generations. Figure 4 depicts CAR-T mechanist of action.

Gliomas, including glioblastoma, bring a unique therapeutic challenge. Their location in the ‘immune-protected’ central nervous system (CNS) means that systemic treatments have the added challenge of navigating the blood-cerebrospinal fluid (CSF) barrier and maintaining their efficacy in the CNS. There is a lack of distinct glioma-specific antigens and a wide heterogeneity in their expression, which is a challenge in developing a therapeutic option. It is postulated that certain gliomas, like glioblastoma, can interact with and alter their TME, inducing significant heterogeneity and treatment resistance [5].

Due to the lack of clear-cut targetable antigens in gliomas, several potential antigens have been targeted in clinical trials. These include B7 homolog 3 (B7-H3), Disiloganglioside (GD2), Eph-A2, Eph-A3, IL-13Ra2, HER2, EGFRvIII and Matrix metalloproteinase-2 (MMP-2).

### B7 homolog 3

B7-H3, also known as CD276, is a member of the B7 family of immune checkpoint inhibitory molecules. It exerts an inhibitory influence on immunoregulation. It is selectively expressed to a large degree in tumours, like gliomas, and in cells in the TME compared to normal cells in the body. It has also been postulated to play a role in tumour proliferation and treatment resistance [6]. It is expressed in 60%–90% of gliomas in clinical studies [7]. Due to its high degree of expression in gliomas compared to normal brain tissue, it is identified as a potential marker for targeted CAR-T therapy. Pre-clinical studies demonstrated the efficacy of B7-H3 targeting CAR-T cells *in vitro* in mouse models [8].

Vitanza *et al* [9] published updated phase I data in 2025 and included patients with diffuse intrinsic pontine glioma (DIPG) aged 1–26 with a median of 6 years. They had a median OS of 10.7 months (range 0.6–45.8 months) on treatment. Given the aggressive nature of DIPG and its median survival being 8–11 months, a 3-year 40% survival for DIPG patients without a prior progression with B7-H3 CAR-T therapy is remarkable. A case report [10] with B7-H3 CAR-T therapy demonstrated a rapid and dramatic reduction in size of recurrent glioblastoma multiforme (GBM) but this response was short-lived with another recurrence at 50 days after the first infusion.

### Disiloganglioside

GD2 is a glycosphingolipid with a sugar chain, found in larger quantities on the cell-surface of CNS tumours, including GBM and DIPG. It is expressed minimally in other CNS tissue. While gangliosides are generally widely expressed in normal tissue, GD-2 specifically is a tumour-associated cell-surface marker and ‘positivity’ for GD2 has been associated with the development of malignant phenotypes, while cell-cycle dysregulation [11]. GD2 positivity has been noted in 50%–90% of malignant gliomas [12]. GD2-targeting CAR-T cells demonstrated efficacy against GBM and DIPG in pre-clinical studies in *in-vitro* models [13–15].

Phase 1 data by Liu *et al* [16] with 4SCAR-T targeting GD2 in GD2-positive GBM showed a median OS of 10 months (3–24 months) in 8 evaluable patients. 3 of them achieved a partial response (PR), 1 with stable disease (SD) and 4 had progressive disease (PD). 4 patients were still alive at 12 or more months after receiving the CAR-T infusion with the longest being 24 months at the time of publication. 4 out of the 8 patients had survival of 23 months or longer (3 still alive at time of censoring).

### EphA2 and EphA3

Eph receptors comprise the largest group of tyrosine kinase receptors and are divided further into A or B depending on the extracellular domain. They play a role in signal transduction pathways in cells affecting growth, differentiation and migration. EphA2 has been demonstrated to be elevated in 90% of cells of GBM but was not found to be so in normal brain tissue; additionally, it was expressed in a discordant manner to its associated ligand ephrinA1 in GBM, thus possibly demonstrating a role in the pathogenesis of this tumour [17]. Data from Lin *et al* [18] of a first in-human trial for EphA2-directed CAR-T cells in 3 patients showed 1 SD and 2 PDs with an OS of 86 to 181 days reported.

EphA3 has been shown to be highly expressed in cells implicated in the initiation of gliomas and is postulated to be playing a role in maintaining these cells in a state of low differentiation, which is important in the pathogenesis of cancer [19]. EphA3 CAR-T cells showed efficacy against GBM in preclinical studies [20].

### IL-13Ra2

IL-13 is an immunoregulatory cytokine. The IL-13 receptor is associated with the IL-13Ra2 ligand chain, noted to be highly expressed in GBM and very low in normal tissue; it is associated with the mesenchymal subtype and portends a poor prognosis. IL-13Ra2 targeting CAR-T cells differ from most other CARs in that their extracellular antigen recognition domain is a naturally occurring IL-13 ligand rather than the scFv found in other CARs.

An initial off-label use of IL-13Ra2-specific CAR-T cells in GBM was reported by Brown *et al* [21]. Six patients with unresectable recurrent GBM with a mean age of 56.2 (36–66) received this family of CAR-T cells via intracerebral catheter without any dose-limiting toxicities; however, it was minimally in blood detected at the 10-month mark. Median survival was 2.9 months, with the longest being 11.3 months.

The expanded, phase I trial by Brown *et al* [22] used IL-13Ra2 CAR-T cells in recurrent, high-grade gliomas in a heavily pre-treated population and with 41 of 58 IDH-wild type. Delivery was either intracranial, intra-tumoural or both. Out of 58 evaluated patients, 35% had Grade 3 or worse toxicities, 29 (50%) patients achieved SD or better, 2 (3.4%) achieved a PR, 1 (1.7%) achieved a CR. The overall cohort had a median OS of 8 months, the population with recurrent GBM had a median OS of 7.7 months. Subset analysis was done stratifying patients by high or low/no CD3 infiltrates in pre-treatment tumour samples. For all recurrent high-grade gliomas, intermediate/high CD3 infiltrates showed a median OS of 11.2 months (10.1–34.7) compared to low/no CD3 median OS of 6.5 months (4.8–9.7) in this study. For recurrent GBM, intermediate/high CD3 had a median OS of 10.3 months (9.2-NA) and low/no CD3 had an OS of 6 months (4.6–8.0). Thus, the higher rates of CD3 positivity in pre-treatment samples correlated to improved outcomes with IL-13Ra2 CAR-T therapy.

Bagley *et al* [23] have reported interim phase 1 data on using bivalent CAR-T cells targeting EGFR and IL-13Ra2 delivered intrathecally in six patients. All six patients experienced immune effector-cell associated neurotoxicity syndrome (ICANS) and 3 SDs at the time of reporting.

### HER2

HER2 is a tyrosine kinase receptor expressed in epidermal tissue across the body but upregulated in tumours, including GBM. An ongoing trial, BrainChild-01 by Vitanza *et al* [24], reported promising phase I data on HER2 targeting CAR-T therapy in relapsed/refractory CNS tumours like gliomas in the pediatric/young adult population. A landmark study by Ahmed *et al* [25] with 17 patients demonstrated a median OS of 11.1 months (95% CI, 4.1–27.2 months) from first infusion and 24.5 months (17.2–34.6 months) from diagnosis. 5 of the 17 patients survived for greater than 20 months after infusion. 3 patients were alive without any evidence of progression at 24–29 months.

### EGFRvIII

EGFRvIII is a specific variant of EGFR that is selectively found in a proportion of GBM cases and not expressed in normal tissue. This allows EGFRvIII to act as a target for selective CAR-T cells against GBM. However, trials using EGFRvIII CARs did not demonstrate desired efficacy. The reasons for this were likely twofold, first these CARs only targeted this single antigen, which, while selective for GBM, was not as frequently upregulated as other antigenic targets discussed in this review, and second, EGFR-wild type remains the most frequently upregulated variant of EGFR in these tumours. To combat this, EGFRvIII CARs were engineered to secrete T-cell engaging antibody molecules (TEAMs) with action against wild-type EGFR.

Goff *et al* [26] reported a phase I trial of EGFRvIII targeting CAR-T therapy in patients with recurrent GBM expressing EGFRvIII. 18 patients were included, median PFS was 1.3 months (1.1–1.9 months) and median OS was 6.9 months (2.8–10 months), which did not demonstrate meaningful efficacy.

Bagley *et al* [27] noted the upregulation of PDL-1 in the TME when using EGFRvIII CAR-T therapy; based on this, a trial of combining EGFRvIII CAR-T therapy with anti-PD1 checkpoint inhibitor pembrolizumab. In this 7-patient study, the median PFS was 5.2 months (90% CI 2.9–6.0 months) and the median OS was 11.8 months (90% CI 9.2–14.2 months).

It was hypothesised that the responses to these CARs may not be as expected, possibly due to the generally higher ratio of wild-type EGFR compared to EGFRvIII. An interesting trial by Choi *et al* [28] used EGFRvIII targeting CARs in conjunction with TEAMs for action against wild-type EGFR. This 3-patient study demonstrated rapid and strong responses; however, the responses did not last long in 2 out of 3 patients, thus requiring optimisation in durability.

### Matrix metalloproteinase-2

MMP-2 acts as a proteolytic enzyme and has a role in breaking down extracellular tissue. It is upregulated in tumours and also plays a role in the development of metastasis. Interestingly, the TME of gliomas modifies MMP-2 in a way that a naturally occurring peptide in the venom of scorpion species Leiurus quinquestriatus, chlorotoxin (CLTX), binds to MMP-2 on the surface of gliomas but not of normal brain tissue. This allows CLTX to act as an antigen recognition domain on the surface of CAR-T cells targeting gliomas. A phase 1 study [29] is recruiting patients at the time of this report.

## Results

Key clinical trials for CAR-T in recurrent gliomas are described below. Target, patient demographics and performance status are described in Table 1. Outcome data, including OS and/or PFS, alongside adverse effect data, are included in Table 2.

Every trial thus far has been in a population with an adequate performance status, either Karnofsky greater than 50 or Eastern cooperative oncology group (ECOG) 0–1 for recurrent gliomas after first-line treatment. Most trials were able to deliver the engineered CARs intracranially. Lifespan of the CARs was over 3–4 weeks in most studies with some studies demonstrating detectable copies as far as 7 months out. These phase I trials were balancing efficacy versus rates of grade 3 or worse adverse events, CRS and ICANS. Initial glance would suggest that glioma’s demonstrating EGFRvIII and IL-13Ra2 had poorer outcomes as compared to B7H3 and GD2, thus the specific mutation affects outcome even with CAR-T cell therapy. Novel and innovative approaches like bi-target CARs, addition of TEAMs and fourth generation ‘armoured’ CAR-T cell therapy will be needed to tip the scale towards efficacy versus toxicity from this treatment. Phase II/III and long term follow up data will be required to demonstrate survival benefit, toxicities and long-term adverse effects such as secondary primary malignancies, which are beginning to emerge with CAR-T therapy in hematologic malignancies.

## Conclusion

Recurrent high-grade gliomas present a unique therapeutic challenge due to their aggressive nature, immune-protected location and ability to interact with the TME. Several antigenic targets are being explored as CAR-T cell therapy, with phase I trials showing relatively promising data in this setting. For patients with an adequate performance status and tumour expressing a targetable cell marker, referral for enrollment in a clinical trial should be considered in the relapsed/refractory setting for gliomas.

## List of abbreviations

B7-H3, B7 homolog 3 protein; CAR, chimeric antigen receptor; CAR-T, Chimeric antigen receptor therapy; CCNU, Lomustine, CNS, central nervous system; CLTX, chlorotoxin; CRS, Cytokine release syndrome; DIPG, diffuse intrinsic pontine glioma; ECOG, Eastern cooperative oncology group; GD2, Disiloganglioside; ICANS, Immune effector-cell associated neurotoxicity syndrome; ICT, intratumoural; ICV, Intracerebroventricular; IV, intravenous; MMP-2, Matrix metalloproteinase 2; OS, overall survival; PD, progressive disease; PR, partial response; scFv, single-chain variable fragment; SD, stable disease; TEAMs, T-cell engaging antibody molecules; TME, tumour microenvironment; TIAN, tumour inflammation-associated neurotoxicity.

## Conflicts of interest

None.

## Funding

No funding.

## Figures and Tables

**Table 1.** Demographic information for key CAR-T trials in gliomas over the past 5 years.

CAR target	Study (year)	Phase	No of Patients (n)	Patient selection criteria	Age (years)	Gender (% Male)	Lines of prior treatment	PS
B7-H3	Vitanza *et al* [9] 2025	1	21	Age >1, <26; DIPG diagnosis; adequate PS and organ function	Median 6 (2–22)	9/23 (39%)	≥1	100% Karnofsky ≥60
GD2	Majzner *et al* [30] 2022	1	3	Age 2–30; H3K27M-mutated DIPG restricted to brainstem	5–25	1/3 (33%)	1–2	100% Karnofsky ≥60 or ECOG 0–1
GD2 (4SCAR-T)	Liu *et al* [16] 2023	1	8	GD2 positive GBM	4 adult (29–63); 4 pediatric (3–6)	4/8 (50%)	1–3	-
EphA2	Lin *et al* [18] 2021	1	3	Recurrent EphA2-positive GBM	30–45	2/3 (67%)	2	-
IL-13Ra2	Brown *et al* [21] 2022	1	6	Age 18–70; progressive/recurrent grade III or IV malignant glioma expressing IL13Rα2 and required ongoing dexamethasone therapy	56.2 + 10.7 (range: 36–66)	4/6 (67%)	1–4	100% Karnofsky ≥60
IL-13Ra2 and EGFR	Bagley *et al* [23] 2024	1	6	Age ≥18; recurrent GBM IDH-wild type and presence of EGFR	33–71	6/6 (100%)	2–3	100% Karnofsky ≥60
IL-13Ra2	Brown *et al* [22] 2024	1	65	Age 18–75; progressive/recurrent grade 3 or 4 malignant glioma, IL-13Ra2+, adequate PS and life expectancy	Median 49 (16–71)	40/65 (62%)	≥2	100% Karnofsky ≥60
HER2	Vitanza *et al* [24] 2021	1	3	Age 15–26; refractory/recurrent HER2+ CNS tumor; adequate PS; presence of CSF catheter	16–26	2/3 (67%)	1–4	100% Karnofsky ≥60
HER2	Burger *et al* [31] 2023	1	9	Recurrent HER2+ GB	30–70	7/9 (77%)	≥2	100% Karnofsky ≥50
EGFRvIII	Goff *et al* [26] 2019	1	18	Adult patients with recurrent EGFRvIII+ GBM	43–66	15/18 (83%)	3-6	100% Karnofsky ≥60
EGFRvIII (and PD-1)	Bagley *et al* [27] 2024	1	7	Adult patients with EGFRvIII GBM; MGMT unmethylated; no prior systemic therapy; adequate PS	56–76	5/7 (71%)	1	100% ECOG 0–1
EGFRvIII and EGFR wt	Choi *et al* [28] 2024	1	3	Adults with recurrent EGFRvIII+ GBM	57–74	2/3 (67%)	2–3	-

**Table 2.** Clinical outcome information for key CAR-T trials in gliomas over the past 5 years.

Trial and Target	Procedure of CAR-T	Efficacy	IDH status	Other mutations	Lifespan, amplification	Grade 3 or worse adverse events	CRS/ICANS
B7-H3 [9]	ICV	PR = 1/18 (6%); SD = 15/18 (83%); PD = 2/18 (11%), Median OS 10.7 months (0.6–45.8)	1 IDH1	1 H3F3A K27M 1 BRAF	Peak detection at week 3	11	CRS, ICANS not reported
GD2 [30]	1st dose IV; Subsequent doses ICV via Ommaya	Best response PR = 3 (100%)	0	3/3 H3K27M		2/3	CRS 3/3, ICANS 1/3, TIAN 3/3
GD2 [16]	IV, IV+ICV	PR = 4/8 (50%); SD = 1/8 (13%); PD 3/8 (38%)	8/8 IDH1/2 wildtype	7/8 UM MGMT	Expand 1–3 weeks, persisted in peripheral blood at low levels after	4/8	CRS, ICANS not reported
EphA2 [18]	IV	SD = 1/3 (33%); PD = 2/3 (67%); OS 86–181 days	-	3/3 EphA2	Peaked days 7–13, detectable beyond 28 days	0/3	CRS 2/3; ICANS not reported
IL-13Ra2 [21]	ICV via Rickham catheter	Median OS 2.9 months	-	6/6 expressed IL-13Ra2	-	0/6	No CRS, ICANS
IL-13Ra2 and EGFR [23]	ICV via Ommaya	3/6 (50%) SD	6/6 IDH-wild type	6/6 EGFR amplification; 1/6 EGFR R108K; 2/6 EGFRvIII; 4/6 UM MGMT, 2/6 Methylated MGMT	Amplification days 1–7	11	CRS 6/6; ICANS 6/6
IL-13Ra2 [22]	ICV, ICT, ICV+ICT	SD = 29/58 (50%); PR = 2/58 (3%); CR = 1/58 (2%)	41/58 IDH-wild type	58/58 IL-13Ra2	Detected in CSF >7 days post infusion in a subset of patients	23	Not reported
HER2 [24]	ICV	SD = 1/3 (33%); PD = 2/3 (67%)	-	3/3 HER2	-	0	Not reported
HER2 [31]	ICV via local injection	SD = 5/9 (56%); PD = 4/9 (44%)	9/9 IDH wild-type	5/9 MGMT+, 9/9 HER2	-	7	0 CRS, ICANS
EGFRvIII [26]	IV	PFS 1.3 months (1.1–1.9); OS 6.9 (1.8–10.0)	-	18/18 EGFRvIII	1 month in 14 patients, 3 months in 5 patients	18/18	CRS 1/18, ICANS 0
EGFRvIII and PD-1 [27]	IV	Median OS 11.8 months (9.2–14.2); median PFS 5.2 months (2.9–6.0)	-	18/18 EGFRvIII	CAR copies detectable in all at 2 months, longest 7 months	5	0 CRS, ICANS
EGFRvIII and EGFR wt [28]	ICV with Ommaya reservoir	SD = 1/3 (33%)	3/3 IDH wild-type	3/3 EGFRvIII	-	2/3	CRS 3/3, ICANS 3/3; none were Grade 3 or worse

IV- Intravenous, ICV- Intracerebroventricular, ICT- intratumoural, CRS- cytokine release syndrome, ICANS- immune-effector cell mediated neurotoxicity syndrome. TIAN- tumour inflammation-associated neurotoxicity
